# Validation of a Method for Cylindrospermopsin Determination in Vegetables: Application to Real Samples Such as Lettuce (*Lactuca sativa* L.)

**DOI:** 10.3390/toxins10020063

**Published:** 2018-02-01

**Authors:** Ana I. Prieto, Remedios Guzmán-Guillén, Leticia Díez-Quijada, Alexandre Campos, Vitor Vasconcelos, Ángeles Jos, Ana M. Cameán

**Affiliations:** 1 Area of Toxicology, Faculty of Pharmacy, University of Sevilla, C/Profesor García González 2, 41012 Sevilla, Spain; anaprieto@us.es (A.I.P.); ldiezquijada@us.es (L.D.-Q.); angelesjos@us.es (Á.J.); camean@us.es (A.M.C.); 2 Interdisciplinary Centre of Marine and Environmental Research—CIIMAR/CIMAR, University of Porto, Terminal de Cruzeiros do Porto de Leixões, Av. General Norton de Matos s/n, 4450-208 Matosinhos, Portugal; amoclclix@gmail.com (A.C.); vmvascon@fc.up.pt (V.V.); 3 Department of Biology, Faculty of Sciences, University of Porto, Rua do Campo Alegre, 4069-007 Porto, Portugal

**Keywords:** cylindrospermopsin, UPLC–MS/MS, method validation, lettuce

## Abstract

Reports on the occurrence of the cyanobacterial toxin cylindrospermopsin (CYN) have increased worldwide because of CYN toxic effects in humans and animals. If contaminated waters are used for plant irrigation, these could represent a possible CYN exposure route for humans. For the first time, a method employing solid phase extraction and quantification by ultra-performance liquid chromatography–tandem mass spectrometry (UPLC-MS/MS) of CYN was optimized in vegetables matrices such as lettuce (*Lactuca sativa*). The validated method showed a linear range, from 5 to 500 ng CYN g^−1^ of fresh weight (f.w.), and detection and quantitation limits (LOD and LOQ) of 0.22 and 0.42 ng CYN g^−1^ f.w., respectively. The mean recoveries ranged between 85 and 104%, and the intermediate precision from 12.7 to 14.7%. The method showed to be robust for the three different variables tested. Moreover, it was successfully applied to quantify CYN in edible lettuce leaves exposed to CYN-contaminated water (10 µg L^−1^), showing that the tolerable daily intake (TDI) in the case of CYN could be exceeded in elderly high consumers. The validated method showed good results in terms of sensitivity, precision, accuracy, and robustness for CYN determination in leaf vegetables such as lettuce. More studies are needed in order to prevent the risks associated with the consumption of CYN-contaminated vegetables.

## 1. Introduction

Cyanobacteria are a group of primitive microorganisms present in several geographical areas worldwide that are able to grow and proliferate in all aquatic and terrestrial ecosystems, from tropical forests to deserts, oceans, and lakes [1]. Eutrophication and climate change may enhance the bloom of cyanobacteria, increasing the amount of bioactive secondary metabolites in freshwaters, compounds that might pose a risk to humans, animals, and plants [2]. Among these secondary metabolites, the alkaloid Cylindrospermopsin (CYN) is an emerging toxin, originally produced by the cyanobacteria *Cylindrospermopsis raciborskii*, but now exhibiting a cosmopolitan distribution pattern [3]. CYN producers belong to several genera, such as *Aphanizomenon*, *Anabaena*, *Raphidiopsis*, *Lyngbya*, and *Umezakia* [4,5,6,7,8], recorded from a great variety of habitats. CYN is a cytotoxin with the structure of a tricyclic guanidine along with a uracil ring [4].

The mechanisms of the toxic action of CYN in several organs are not totally clarified [1], although evidence suggests that, both in vitro and in vivo, CYN induced a concentration-dependent protein and glutathione synthesis inhibition as well as genotoxicity due to DNA fragmentation [9,10,11,12,13]. Besides, CYN activation by cytochrome P450 seems to be related to its increased toxicity [14], suggesting that the initial compound and its possible metabolites could act by different mechanisms [1]. Oxidative stress is also involved as a mechanism of CYN toxicity in both in vitro [15,16] and in vivo assays [17,18,19]. For consumers protection and to prevent CYN adverse effects, 0.03 µg kg^−1^ of body weight (b.w.) has been settled as the provisional Tolerable Daily Intake (TDI) for this toxin [20].

In comparison to mammals, the amount of studies that investigated the physiological and biochemical effects of CYN on diverse plants is scarcer. These studies have been reviewed by Corbel et al. (2014) [21] and, more recently, by Machado et al. (2017) [2]. These effects include alterations in growth [22,23,24,25], germination, and development [26,27], oxidative stress [28,29], decreases in chlorophyll [30], chromosomal aberrations [31], changes in mineral content [25,29] and in the proteome [32].

Generally, humans can be exposed to cyanotoxins by the oral route through several pathways: when drinking water contaminated with cyanotoxins, when consuming fish, vegetables, crops and even food supplements susceptible to contain cyanotoxins, or accidentally by ingestion of water while performing recreational activities [1]. The ability of cyanotoxins such as microcystins (MCs) to accumulate in the tissues of a wide range of agricultural crops has been described and reviewed [2,21,33], although only a few studies have been carried out on the leaves of edible plant species, such as lettuce *(Lactuca sativa)* [34,35,36].

However, in spite of the human health risks associated with CYN, less attention has been given to its bioaccumulation in edible agricultural plants compared with MCs [37], which is considered a public health concern, given CYN's high water-solubility and its ability to transfer to higher trophic levels [38]. This bioaccumulation may be possible because of the absorption of toxins by plants if surface water contaminated with cyanotoxins is used in agriculture, thus posing risks for food safety. In this sense, field studies have shown a broad range for concentrations of CYN in waters worldwide, although ecologically relevant CYN levels potentially used for the irrigation of vegetables ranged between 5 and 100 µg L^−1^ [19]. At these concentrations, the ability of CYN to enter the food web by this last route has been minimally studied. To fill this gap in knowledge, advances in this field could provide helpful information for public health, concerning the bioaccumulation of CYN in plants and how water resources should be used to minimize crop contamination. White et al. (2005) [39] suggested that the aquatic macrophyte *Hydrilla verticillata* might adsorb CYN in the plant cell walls instead of taking up the toxin through the cells. CYN transdermal absorption could represent an important route of plant uptake because of CYN high presence in dissolved form in the environment [40,41]. Silva & Vasconcelos (2010) [27] observed that the roots of *L. sativa*, *Phaseolus vulgaris*, and *Pisum sativum* accumulated higher concentrations of CYN in comparison to the stems. Additionally, Prieto et al. (2011) [28] found accumulation of CYN in plants of *Oryza sativa* exposed to 2.5 μg CYN L^−1^ from an extract, showing a higher amount of the toxin in the roots, compared to the leaves. CYN was also detected in various *Brassica* vegetables (roots and leaves) after exposure to a CYN-containing extract, and this accumulation seemed to depend on the concentrations applied to the roots (18–35 μg L^−1^), with the levels of CYN ranging from 10 to 21% in the leaves [42]. Recently, a study showed that the bioaccumulation of CYN in lettuce *(L. sativa*) and arugula (*Eruca sativa*) depends indirectly on the exposure concentration, and also on time and on the species [37]. For example, after 7 days of exposure, the CYN concentrations measured in lettuce leaves were 8.29, 4.19, and 3.78 µg CYN kg^−1^ for exposure concentrations of 3, 5, and 10 µg CYN L^−1^, respectively. In the case of arugula, these values were 11.49, 10.41, and 9.47 µg CYN kg^−1^ for the three levels of exposure.

Fresh products such as lettuce, spinach, cabbage, and sprouts are processed minimally. Their consumption has increased over the last decade, maybe because of their high nutritional value, changes in social eating habits, and wider accessibility [43]. Some recommendations by international organizations and limit values have been set worldwide in order to prevent or manage the potential effects on human health induced by the exposure to CYN under different scenarios [1]. However, no recommendations or limits have been established so far in the case of vegetables, despite the results demonstrating the risk of ingesting CYN through contaminated vegetables [37]. Following the recommendations of the European Food Safety Authority (EFSA), there is a need to develop analytical methods for sample preparation and detection of cyanotoxins in complex matrices such as food items [44]. In this sense, some methods for cyanotoxins analysis in different matrices such as natural blooms, cyanobacterial strain cultures, and biological samples, are reported in the literature and use Liquid Chromatography (LC)–Mass Spectrometry techniques [36,45,46,47,48,49,50,51], whose criteria and applications have been recently reviewed by Caixach et al. (2017) [52]. Concerning CYN, no validation procedures with robustness tests have been developed in vegetables compared to other matrices such as waters, cyanobacterial cultures, or fish tissues [50,51,53]. In fact, and specifically in relation to the matrices assayed in the present study, CYN has been detected in *Brassica* vegetables, lettuce, and arugula by ELISA and LC–MS/MS [37,42], but these methods have not been validated. Ultra-performance liquid chromatography–tandem mass spectrometry (UPLC–MS/MS) allows excellent specificity and sensitivity for cyanotoxins detection and quantification in waters and also in more complicated matrices, becoming the technique of choice for these purposes [54,55,56].

For these reasons, the present work aimed for the first time to develop and validate an analytical method based on UPLC–MS/MS for the extraction and quantification of CYN in edible vegetables samples (*Lactuca sativa* L.). The method has been optimized and validated according to international guides [57,58]. The present procedure has been designed for the routine determination of CYN in leaves of edible lettuces intended for human consumption, for prevention and risk assessment purposes.

## 2. Results and Discussion

Before testing the efficiency of the CYN extraction method, the UPLC–MS/MS method was setup for this purpose. For this, commercially purchased CYN standard solutions were tested, acquiring mass spectra and adjusting the mobile phase strength, as previously performed in our laboratory [55,56]. The spectrum was obtained on collision of *m*/*z* 416, corresponding to the pseudomolecular ion [M + H]^+^. The selected transitions for the quantitation and confirmation of the analyte CYN were 416.2/194.0 and 416.2/176.0, respectively. Specifically, the signals at *m*/*z* 194 and 176 correspond to the loss of SO_3_ and H_2_O from the fragment ion at *m*/*z* 274, corresponding to the loss of the [6-(2-hydroxy-4-oxo-3-hydropyrimidyl)] hydroxymethinyl moiety from the CYN structure [59].

### 2.1. Calibration Study

**Figure 1:**
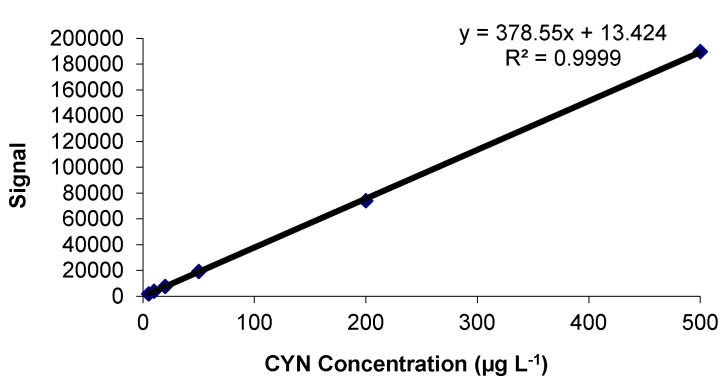
Calibration curve function proposed with a linear range within 5 to 500 µg Cylindrospermopsin (CYN) L^−1^.

A prerequisite for carrying out a quantification is to establish a calibration function for the final measuring instrument [60]. The response as a function of concentration was calculated from CYN standards prepared in fresh lettuce leaves extracts and was measured by a calibration curve including six points within the linear range 5–500 µg L^−1^ (equivalent to 5–500 ng CYN g^−1^ f.w. lettuce). The regression equation obtained was *y* = 378.55*x* + 13.424 (*r*^2^ = 0.9999) (Figure 1).

#### 2.1.1. Linearity and Goodness of the Fit

**Figure 2:**
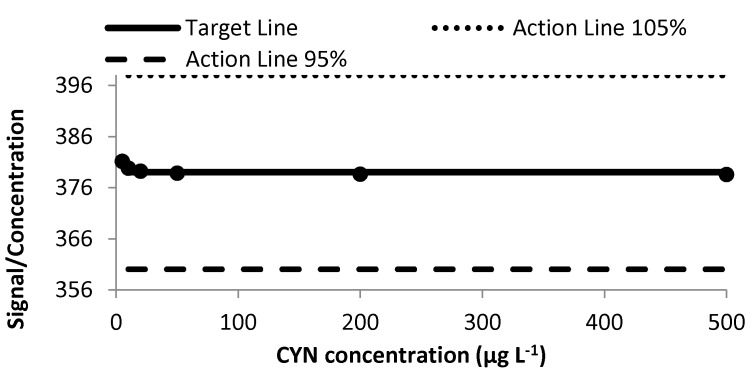
Response linearity in lettuce according to Huber plot.

Six different concentrations of CYN were spiked in blank extracts of lettuce leaves (5–500 ng CYN g^−1^ f.w.), and the samples were submitted to the proposed method. The response linearity was set according to Huber (1998) [61] by plotting each concentration assayed against its respective response factors (signal response/analyte concentration). The target line represents the median of the response factors obtained and has zero slope, while the two parallel horizontal lines represent 0.95 and 1.05 times the median value (Figure 2). The linear range of the method applies to the full range studied because no intersections with the lines were found. Moreover, with the same signal responses, the corresponding ANOVA of the regression line was performed, indicating a lack-of-fit *F* ratio of 5.39 compared to a tabulated *F* value of 19.4, and, consequently, the calibration function can be considered as linear.

#### 2.1.2. Limits of Detection and Quantitation

The limits of detection (LOD) and quantitation (LOQ) are based upon the variability of the blank and are calculated according to the equation Y_LOD or LOQ_ = Y_blank_ + *n*S_blank_, where Y_blank_ is the mean value of 10 blank signals, S_blank_ its corresponding standard deviation, and *n* is a constant (3 for LOD and 10 for LOQ). The LOD and LOQ obtained were 0.22 ng CYN g^−1^ f.w. and 0.42 ng CYN g^−1^ f.w (equivalent to 0.22 and 0.42 µg L^−1^), respectively. Both limits are below the proposed guideline value of 1 µg L^−1^ for CYN in waters and are lower than the LOD and LOQ values of 0.5 and 80 µg L^−1^, respectively, reported in the same matrix, i.e., lettuce leaves, by Cordeiro-Araújo et al. (2017) [37]. Moreover, the LOD and LOQ found in the present study would permit the quantification of CYN at the concentrations detected in other studies carried out with *Brassica* vegetables (2.71 ng g^−1^) [42], as well as with lettuce and arugula (3.76 and 5.5 ng CYN g^−1^ f.w, respectively) [37].

#### 2.1.3. Precision

Precision is a measure of the closeness of agreement between mutually independent measurement results obtained under specified conditions. It is generally dependent on the analyte concentration [57], and its measure includes three concepts: repeatability, intermediate precision, and reproducibility [62]. Repeatability is a measure of variability in the results in the case that the measurements are performed on the same material by a single analyst employing the same method and equipment over a short timescale, while intermediate precision estimates the variation in the results in the case that the measurements are made on the same material in a single laboratory applying the same method over an extended timescale, and therefore refers to more variable conditions compared to repeatability. On the other hand, reproducibility is a measure of the variability in the results when the measurements are performed in different laboratories and, therefore, of what is expected to represent the greatest variation in the results [57].

The values of repeatability (within-day and between-day, Sw and S_B_), intermediate precision (intralaboratory reproducibility, S_IP_), and S_IP_ relative standard deviations (%RSD_IP_) were calculated analyzing three replicates of lettuce leaves extracts spiked with standard CYN solutions at different concentrations (20, 200 and 500 µg L^−1^) on the same day, following the ICH guidelines, and over a period of three consecutive days. Considering three different days as the main source of variation, the estimations of the precision parameters were obtained by performing an analysis of variance (ANOVA) for each validation standard according to González and Herrador (2007) [63] and González et al. (2010) [64] (Table 1). According to the Eurachem/CITAC Guide [57] our results show that the values of repeatability (S_W_) represent the smallest variation. Moreover, the corresponding %RSD_IP_ were compared with the acceptable RSD percentages obtained from the AOAC guidelines (2016) [58]. Our results, at the three concentration levels considered, were lower than or of the same order of the tabulated %RSD_AOAC_ (15–21% for 20 µg L^−1^, and 11–15% for 200–500 µg L^−1^).

#### 2.1.4. Trueness and Recovery

Trueness is the closeness of agreement between a test result and the accepted reference value of the property being measured. Trueness is stated quantitatively in terms of “bias”, with smaller bias indicating greater trueness [65], and can be expressed as the recovery obtained for each validation standard assayed. The total recovery for each validation standard (20, 200, or 500 µg L^−1^) was obtained as the ratio between the observed estimation of the standard concentration and the true value, expressed as fraction or as percentage. The recoveries (%) obtained were 104 ± 2 for 20 µg CYN L^−1^, 90 ± 5 for 200 µg CYN L^−1^, and 85 ± 3 for 500 µg CYN L^−1^. The suitability of these values was checked by comparison with the published acceptable recovery percentages depending on the analyte concentration, according to AOAC (2016) [58]. Specifically, taking into account the CYN concentration of the three validation standards, the acceptable recovery range (%) according to AOAC could vary within 80% and 110%. Consequently, in terms of recoveries, the proposed method can be considered as acceptable.

#### 2.1.5. Ruggedness Study

The ruggedness (sometimes also called robustness) is a parameter that has often been required in the validation studies of analytical methods and to assess the vulnerability of factors in the analytical methods optimized by using the chemometric tools [66]. These studies evaluate a method’s capacity to remain unaffected by small variations in some method parameters [57]. The strategy when performing the present ruggedness study was based on the method suggested by Youden (1967) [67], according to González and Herrador (2007) [63]. Three deliberate variations in the extraction procedure of the method were chosen: F1, i.e., time for the sample to pass through the cartridge, F2, i.e., the water volume for washing the cartridge, and F3, i.e., the elution volume of DCM/MeOH. The levels were coded as specified in Table 2. By combining these parameters, the resulting eight different possibilities were tested. To determine whether the variations in an experimental setting have a significant effect on the results, a significance *t* test is applied by González and Herrador (2007) [63], and the *t* values (Fx) are compared with the 95% confidence level two-tailed tabulated value (*t*_tab_) with eight degrees of freedom (d.f.) derived from the precision study for each concentration tested. The effect of every factor is estimated as the difference of the average result obtained at the level +1 and that obtained at the level −1. The two-level full factorial design is the most efficient chemometric tool for ruggedness evaluation [66]. In the present work, the experiments were performed using lettuce leaves spiked with an intermediate validation standard of 200 µg CYN L^−1^ (equivalent to 200 ng g^−1^ f.w.), and each factor was analyzed by triplicate on three different days obtaining eight d.f. The *t* values obtained were F1 = 1.36, F2 = 0.297, and F3 = 0.208, being in all cases lower than the tabulated value (*t*_tab_ = 2.306). These results showed that the present validated method can be considered as a robust method against the three factors considered at the levels fixed in the study.

### 2.2. Application of the Proposed Method to Edible Vegetables Exposed to CYN

**Figure 3:**
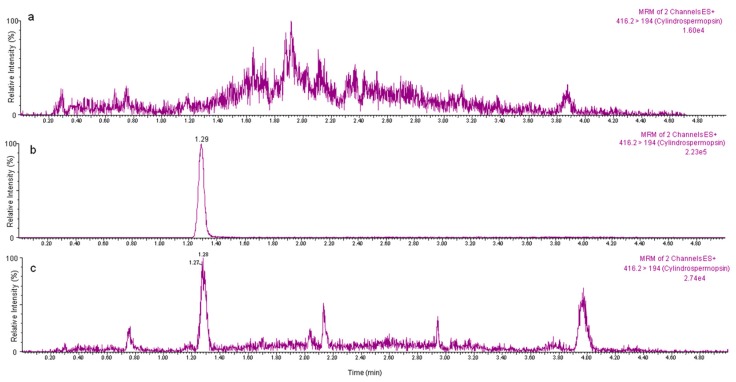
MRM chromatograms by UPLC–MS/MS of (**a**) an extract from a control lettuce sample without the toxin; (**b**) a CYN standard solution (20 µg CYN L^−1^); (**c**) an extract from a lettuce sample exposed to a solution of purified CYN (10 µg CYN L^−1^) from a *Chrysosporum ovalisporum* (LEGE X-001) culture.

The validated method was finally applied to quantify the unconjugated CYN concentrations in the leaves of the control lettuce (*n* = 5) and of lettuces grown in a soil-free hydroponic cultivation system (*n* = 5), following the experimental conditions indicated (‘Experimental exposure of vegetables and application of the validated method’ section). CYN was not detected in the control lettuces, and 2.37 ± 0.80 ng CYN g^−1^ f.w. was detected in the experimental lettuces exposed to 10 µg CYN L^−1^ (Figure 3). The CYN uptake obtained (23.7%) for lettuce agrees with the previous values presented by Kittler et al. (2012) [42] for kale (*Brassica oleracea* var. *sabellica*), corresponding to 10–15%, and for vegetable mustard (*Brassica juncea*) (12–21%) exposed to 18.2–35.5 µg CYN L^−1^, using a hydroponic cultivation system. Similarly to these authors, in this work we have demonstrated that CYN is absorbed by the roots of lettuces and transported to the leaves and that the adsorptive effects of the soil particles have not been taken into account. Previously, the transfer of CYN to the upper part of a plant (stem) was also observed in lettuce (*L. sativa*), bean (*Phaseolus vulgaris*), and peas (*Pisum sativum*) exposed to CYN-contaminated water obtained by exposure to a *C. raciborskii* strain (0.57–57 µg CYN L^−1^) [27]. Prieto et al. (2011) [28] detected CYN in the leaves of *O. sativa* plants (measured by ELISA test) suggesting the uptake of CYN by the roots and its further translocation to other organs of the plant. Moreover, the only data found in the scientific literature reporting CYN levels in lettuce leaves, obtained by a non-validated method in this matrix by Cordeiro-Araújo et al. (2017) [37], were also similar to those found in the present work. These authors found a concentration-dependent CYN bioaccumulation in lettuce, and the mean value reported after exposure to 10 µg CYN L^−1^ for 7 days was 3.78 ± 0.25 ng g^−1^. Our results confirm the uptake of CYN in edible vegetables exposed to low and ecologically relevant concentrations of the toxin, posing potential public health consequences.

In comparison to other cyanotoxins, MC-LR showed a similar pattern of accumulation in agricultural plants [2]. Thus, in lettuce sprayed with MCs (ranged between 0.62 and 12.5 µg L^−1^ for 15 days), the concentrations found in the foliar tissues were 8.31–177.8 µg MCs kg^−1^ f.w. [34], higher than the values obtained by Crush et al. (2008) [68]. The accumulation of MCs in lettuce had a significant positive correlation with the different exposure concentrations of MCs (MC-LR and MC-RR) and could imply serious human health risks when consuming contaminated vegetables [35], although the possibility of MC-LR decontamination of lettuce leaves has been demonstrated [36].

Assuming a human consumption of 40 g of vegetable per day for an adult of 60 kg (equivalent to 0.65 g kg^−1^ b.w. day) [2,37], the ingestion is 1.58 ng CYN kg^−1^ b.w. per day (2.37 ng CYN/g f.w. × 40 g vegetable/60 kg b.w.). Taking into account the recommended TDI for CYN of 0.03 µg kg^−1^ per day, the consumption of lettuce leaves in this case (1.58 ng CYN kg^−1^ b.w. per day) would account for 5.3% of the TDI of this toxin. For a more detailed exposure assessment in relation to different age groups, we took into account the data provided by the EFSA, Comprehensive European Food Consumption Database [69] (Table 3) for normal and high consumers (95th percentile). Thus, it was evidenced that the CYN uptake in adults (mean ranged between 0.07–1.54 ng CYN kg^−1^ b.w.day) was similar to that reported by Cordeiro-Aráujo et al. (2017) [37]. Moreover, the age group with a higher CYN exposure would by the elderly (65–75 years) with a potential CYN exposure of 0.17–12.77 ng CYN kg^−1^ b.w.day (mean values) or of 0.57–70.53 for high consumers. These values could contribute to the TDI of CYN in a range of 0.55–42.58% and 1.90–235.1%, respectively. Therefore, in this group age health risks after consumption of CYN-contaminated lettuce cannot be excluded. These findings highlight the need to develop adequate validated methods for an accurate determination of CYN in order to know and to prevent the health risks for the consumers.

## 3. Conclusions

A UPLC–MS/MS method has been developed and validated for the first time in order to detect unconjugated CYN in the leaves of lettuce (*L. sativa*), showing acceptable sensitivity, reproducibility, accuracy, and robustness. Its linearity, the LOD and LOQ, recoveries (85–104%), and intermediate precisions obtained (12.72–14.70%) confirm its validation. In addition, this method has been successfully applied to detect CYN at ecologically relevant concentrations in lettuce leaves exposed to CYN, showing that CYN can accumulate in edible vegetables intended for human consumption. Although the TDI was exceeded in most age groups considered, in elderly high consumers health risks after consumption of CYN-contaminated lettuce cannot be excluded. These results demonstrate the need and importance of developing accurate analytical methods to monitor CYN in leaf vegetables with the intention of reducing or preventing the associated potential health risks.

## 4. Materials and Methods

### 4.1. Chemicals and Reagents

Cylindrospermopsin standard (95% purity) was purchased from Alexis Corporation (Lausen, Switzerland). CYN standard solutions were prepared in Milli-Q water (100 µg mL^−1^) and further diluted for their use as working solutions (5–500 µg L^−1^). All reagents and chemicals employed in this work were of analytical grade. HPLC-grade methanol, acetonitrile, acetic acid, dichloromethane, and trifluoroacetic acid (TFA) were supplied by Merck (Darmstadt, Germany). Deionized water (418 MΩ cm^−1^ resistivity) was acquired from a Milli-Q water purification system (Millipore, Bedford, MA, USA). BOND ELUT^®^ Carbon cartridges (PGC, 500 mg, 6 mL) were obtained from Agilent Technologies (Amstelveen, The Netherlands, Europe). Reagents for UHPLC–MS/MS were of LC–MS grade; formic acid was supplied by Fluka (Steinheim, Germany) and water and acetonitrile by VWR International (Fontenay-sous-Bois, France). The lettuce samples without CYN were purchased from a local supermarket. In this way, we ensured that they had passed the quality controls required for their human consumption.

### 4.2. Solvent Extraction and Purification Procedures

Preliminary tests were performed to evaluate the influence of the lyophilization process on CYN concentration. For this, 1 mL of a CYN solution (200 μg L^−1^) was added to 1 g of lettuce (fresh weight, f.w.) and the sample was lyophilized (−80 °C). In parallel, 1 mL of the same CYN solution (200 μg L^−1^) was added to 0.05 g of lyophilized lettuce (dry weight, d.w.), corresponding to 1 g of f.w. These procedures were performed in triplicate. Subsequently, CYN was extracted from all the samples to evaluate the influence of these processes. Since no significant differences were observed between the conditions (data not shown), we decided to work with fresh-weight lettuce, once the plants had been exposed to the toxin, as they reach the final consumer.

To develop the extraction and purification procedures, a modified version of the method from Kittler et al. (2012) [42] was performed, adding a purification stage with graphitized carbon cartridges. Briefly, control fresh lettuce leaves (1.05 ± 0.14 g f.w.) were fortified with CYN standard solutions to obtain three concentration levels: 20, 200, and 500 ng CYN g^−1^ f.w. Afterwards, the toxin was extracted with 6 mL of 10% acetic acid; after ultraturrax homogenization for 30 s, the sample was sonicated (15 min) and stirred (15 min). The resulting mixture was centrifuged at 12,000 rpm for 15 min. Once the extract was obtained, a purification step was applied. For this purpose, BOND ELUT^®^ Carbon cartridges were activated with 10 mL of a solvent mixture of DCM/MeOH (10/90) acidified with 5% formic acid and rinsed with 10 mL of Milli-Q water. Then, the sample was loaded, the column was washed with 10 mL of Milli-Q water, and the analyte was eluted with 10 mL DCM/MeOH (10/90) acidified with 5% formic acid. To concentrate the sample, the extract was evaporated in a rotary evaporator and resuspended in 1 mL Milli-Q water, prior to its UPLC–MS/MS analysis.

### 4.3. Chromatographic Conditions

The chromatographic separation was carried out with a UPLC Acquity (Waters, Milford, MA, USA) coupled to Xevo TQS-micro (Waters, Milford, MA, USA) consisting of a triple quadrupole mass spectrometer equipped with an electrospray ion source operated in positive mode. UPLC analyses were performed on a 50 × 2.1 mm Acquity BEH C18 1.7 µm column, at a flow rate of 0.45 mL min^−1^. For chromatographic separation, a binary gradient was used with (A) water and (B) methanol as mobile phases, containing, both of them, 0.1% formic acid (*v*/*v*). The injection volume was 5 µL. The profile for elution was: 0% B (0.8 min), linear gradient to 90% B (2.2 min), 90% B (1 min), and finally 100% B (1 min). Multiple Reaction Monitoring (MRM) was applied, by which the parent ions and fragments ions were monitored at Q1 and Q3, respectively. The transitions for the analyte CYN are 416.2/194.0 and 416.2/176.0. The transition 416.2/194.0 (quan) was chosen for quantitation of CYN, and the transition 416.2/176.0 (target) as confirmatory. The ion ratio were measured in the CYN standard solution and in the lettuce samples as target area/quan area × 100, giving a mean ion ratio of 38.8% with SD of 1.2% for the CYN standard solution (*n* = 10), and a mean ion ratio of 39.6% with SD of 5.1% for CYN in the lettuce samples (*n* = 5) exposed to the toxin. For UPLC–ESI–MS/MS analyses, the mass spectrometer was set to the following optimised tune parameters: Capillary voltage: 3.0 kV, Source Temperature: 500 °C, and source Gas flow: 1000 L h^−1^.

### 4.4. Experimental Exposure of Vegetables and Application of the Validated Method

The lettuce plants (*Lactuca sativa*) were purchased from a local market (Porto, Portugal) as sprouts. The roots were washed with deionized water to remove all remaining soil and prepare the plants for hydroponic cultivation. The plants were introduced into opaque glass jars, and the roots were completely immersed in culture medium [70] at pH 6.5, as described by Freitas et al. (2015) [29]. The plants were acclimated for one week with white fluorescent light (light–dark period of 14–10 h), at a controlled temperature of 21 ± 1 °C until the start of the experiment.

The *Chrysosporum ovalisporum* (LEGE X-001) cyanobacterial CYN-producing strain (CYN+) was isolated from Lake Kinneret, Israel [6] and was grown for biomass production in the Interdisciplinary Centre of Marine and Environmental Research, CIIMAR (Porto, Portugal) as described in Guzmán-Guillén et al. (2014) [71]. The extraction of CYN from the culture was performed according to Guzmán-Guillén et al. (2012) [50] with modifications described in Guzmán-Guillén et al. (2017) [55]. CYN retention time was 9.55 min, and the concentration obtained was 2.14 μg CYN mg^−1^ of lyophilized cells.

After acclimation, five specimens of lettuce were exposed to a solution of CYN extracted from the culture at a concentration of 10 µg L^−1^. In order to do this, the culture medium was changed three times a week for 21 days, adding to the medium the volume of *C. ovalisporum* culture necessary to reach the exposure level (10 μg L^−1^). Moreover, five lettuce plants were included in the experiment as a control group without exposure to the toxin. When the experiment was over, the plants were washed with distilled water, frozen, and lyophilized (Telstar Lyoquest) for CYN extraction, following the validated method proposed in the present study.

### 4.5. Method Validation

The proposed method was validated taking into account the Eurachem (2016), the AOAC (2016), and González and Herrador (2007) guides [57,58,63] for linearity, sensitivity, precision, recovery, and robustness. Three CYN validation standards were employed, performing the measures in triplicate each day for three consecutive days, and these concentrations tried to cover the optimal working range.

One mL of solutions at three different CYN concentrations (20, 200, and 500 µg CYN L^−1^) was added to the plant matrix to obtain 20, 200, and 500 ng g^−1^ f.w. Precision and recovery were obtained by applying a one-factor analysis of variance (ANOVA), as explained in the Results and Discussion section, and then they were compared with tabulated reference values. Moreover, a robustness (ruggedness) study was performed by spiking the matrices with an intermediate validation standard of 200 µg CYN L^−1^ (equivalent to 200 ng g^−1^ f.w.), according to Youden's procedure (1967) [67]. Small, deliberate variations in the following parameters were tested with Student’s t test in order to evaluate the ruggedness of the method: (1) time for the sample to pass through the cartridge; (2) water volume for washing the cartridge; and (3) volume of DCM/MeOH employed for CYN elution.

## Figures and Tables

**Table 1:** Estimations of within-condition repeatability (S_w_), between-condition repeatability (S_B_), intermediate precision (intralaboratory reproducibility, S_IP_), S_IP_ relative standard deviations (%RSD_IP_), and recoveries of CYN at three concentration levels, in three different days, assayed in lettuce samples. Limits of detection (LOD) and quantitation (LOQ) for the lettuce matrix. ^a^ fw: fresh weight. RSD_AOAC_ (%): 15–21% for 20 µg L^−1^ and 11–15% for 200 and 500 µg L^−1^. Acceptable Recovery Range (%) by AOAC: 80–110%.

Analytical Parameters							
CYN Concentration Level (µg L^−1^)	S_W_ (µg L^−1^)	S_B_ (µg L^−1^)	S_IP_ (µg L^−1^)	RSD_IP_ (%)	Recoveries (%)	LOD (ng g^−1^ fw ^a^)	LOQ (ng g^−1^ fw ^a^)
20	1.88	3.72	2.64	12.72	104	0.22	0.42
200	19.25	36.96	26.50	14.70	90		
500	48.79	78.21	60.22	14.20	85		

**Table 2:** Coding rules for the combination of the parameters in the robustness study. F1: time for the sample to pass through the cartridge; F2: water volume for washing the cartridge; F3: elution volume of DCM/MeOH; the *t* values were obtained for each parameter by performing the significance *t* test.

Combined Variables			*t* Values
F1	High (+)	1 min and 15 s	1.36
	Low (−)	1 min	
F2	High (+)	10 mL	0.297
	Low (−)	9.5 mL	
F3	High (+)	10 mL	0.208
	Low (−)	9.5 mL	

**Table 3:** Lettuce consumption data (g kg^−1^ b.w. day) in Europe. Potential CYN exposure in normal and high consumers of lettuce and estimated % TDI. Data obtained from the chronic food consumption statistics (consumers only) of the EFSA, Comprehensive European Food Consumption Database for the food category: Lettuce, excluding Iceberg-type lettuce.

Population Class	Age Range (Years)	Lettuce Consumption (g kg^−1^ b.w. Day)		Potential CYN Exposure (ng CYN kg^−1^ b.w. Day)		Estimated % TDI	
		Mean Range	95th Percentile Range	Mean Range	95th Percentile Range	Mean Range	95th Percentile Range
Children	3–10	0.05–1.15	0.05–3.40	0.12–2.72	0.12–8.06	0.39–9.08	0.39–26.86
Adolescents	10–18	0.07–0.92	0.16–2.78	0.17–2.18	0.38–6.59	0.55–7.27	1.26–24.23
Adults	18–65	0.03–0.65	0.11–1.56	0.07–1.54	0.26–3.70	0.24–5.13	0.87–12.32
Elderly	65–75	0.07–5.39	0.24–29.76	0.17–12.77	0.57–70.53	0.55–42.58	1.90–235.10
Very elderly	>75	0.06–0.75	0.10–1.21	0.14–1.78	0.24–2.87	0.47–5.92	0.79–9.56
